# Not Your Usual Staph: Recurrent Human Infection With the Canine-Associated Staphylococcus pseudintermedius

**DOI:** 10.7759/cureus.100522

**Published:** 2025-12-31

**Authors:** Michael Woo, Anna D Kamuda, Elizabeth Chung, Robert Jellinger, Aahana Gaur, Nino Marzella

**Affiliations:** 1 Internal Medicine, State University of New York Downstate Medical Center, Brooklyn, USA; 2 Pharmacy, Veterans Affairs Medical Center, New York Harbor Healthcare System, Brooklyn, USA; 3 Infectious Disease, Veterans Affairs Medical Center, New York Harbor Healthcare System, Brooklyn, USA; 4 Pharmacy, Long Island University Pharmacy, Arnold and Marie Schwartz College of Pharmacy and Health Sciences, Brooklyn, USA

**Keywords:** antimicrobial resistance, coagulase-positive staphylococcus, dog-to-human transmission, maldi-tof, recurrent infection, skin and soft tissue infection, staphylococcus pseudintermedius, zoonosis

## Abstract

*Staphylococcus pseudintermedius* is a coagulase-positive bacterium commonly found as part of the normal flora in healthy dogs. In clinical settings, infections caused by *S. pseudintermedius* are often misidentified as *Staphylococcus aureus* due to similarities in morphology and the relative rarity of *S. pseudintermedius* as a documented human pathogen. Close contact with dogs has been associated with human infection, but the exact frequency and mechanism of zoonotic transmission remain unclear. Accurate and timely identification of the *Staphylococcus* species is essential for appropriate treatment. We present a case of *S. pseudintermedius* infection likely transmitted from a patient’s household dog. The zoonotic origin of the infection was initially unrecognized, as its clinical significance was misjudged. However, the patient experienced a complicated course with recurrent skin and soft tissue infections, highlighting the diagnostic and treatment challenges of zoonotic *S. pseudintermedius* infections.

## Introduction

*Staphylococcus intermedius* was first described in 1976, and later reclassified as laboratory identification techniques advanced. In 2005, the *Staphylococcus intermedius* group (SIG) was reclassified into three distinct species: *S. intermedius*,* Staphylococcus pseudintermedius*, and* Staphylococcus delphini* [1,2]. Among these, *S. pseudintermedius* has been recognized as an important opportunistic pathogen in animals, particularly domestic dogs [3,4]. Importantly, *S. pseudintermedius* can persist as normal flora on the skin, nares, oral cavity, groin, and anus of healthy dogs. However, it has also been implicated in clinical conditions such as pyoderma, otitis externa, and surgical site infections [3-7].

*S. pseudintermedius* is an emerging zoonotic pathogen that can cause wound infections and skin and soft tissue infections (SSTIs). Similar to Staphylococcus aureus, *S. pseudintermedius* can colonize its host and cause infection when predisposing factors are present, such as immunosuppression, skin barrier disruption, or recent surgical intervention [3,4,6]. Both species can be found in animals and humans. *S. pseudintermedius* is typically transmitted vertically among animals and horizontally to humans. To date, human-to-human transmission has not been clearly established [3-7].

Advances in microbiological diagnostic tools have significantly improved the identification of *Staphylococcus* species. Previously, many gram-positive, coagulase-positive organisms were misclassified as *S. aureus* due to reliance on biochemical testing alone. Matrix-assisted laser desorption/ionization time-of-flight mass spectrometry (MALDI-TOF MS) has revolutionized microbial identification by analyzing protein spectra [8]. This technology has led to increased recognition of *S. pseudintermedius* in both veterinary and human clinical microbiology; yet, the prevalence and clinical significance of *S. pseudintermedius* infection remain poorly established due to the limited number of reported cases. This case is noteworthy because it illustrates the diagnostic challenges posed by *S. pseudintermedius* infection in humans, including recurrent disease, initial misidentification as *S. aureus*, and identifying zoonotic exposure.

## Case presentation

The patient is a 71-year-old Caucasian man with a history of uncontrolled hypertension, atrial fibrillation, stage three chronic kidney disease (CKD), chronic venous insufficiency, stasis dermatitis, right lower extremity (RLE) deep vein thrombosis (DVT), left total knee arthroplasty, and a remote history of intravenous drug use. The patient had a previous negative HIV test and had no immunocompromising conditions besides his stage 3 CKD. His past infectious history includes treated hepatitis C with sustained viral response, septic arthritis, brucellosis, and recurrent bilateral lower extremity (LE) ulcers. He also owned a large dog with which he had frequent close contact.

At the initial presentation, the patient developed a left lower extremity (LLE) wound infection following trauma. He was intermittently febrile and had recently been treated for a chronic prosthetic joint infection. His dog recently had skin infections, though further details regarding presentation and treatment were not obtained. Examination revealed three anterior tibial ulcers with a large area of circumferential erythema, warmth, and serous drainage without purulence. Wound culture grew pansensitive *Enterobacter cloacae* and *S. aureus* susceptible to oxacillin, clindamycin, moxifloxacin, linezolid, trimethoprim/sulfamethoxazole (TMP/SMX), and vancomycin. He was treated with TMP/SMX for 14 days in consideration of the wound culture susceptibilities. Treatment resulted in decreased knee swelling and resolution of fevers.

The patient did not return for any similar complaints regarding a skin infection until three years later, when he presented to the vascular surgery clinic with a painful 2x2 cm left anterior leg ulcer following minor trauma. The patient complained of foot pain, numbness, tingling, and burning, but he did not have any systemic symptoms. The ulcer was surrounded by erythema. He was empirically started on amoxicillin-clavulanate. Wound culture grew *S. pseudintermedius*, which was resistant to clindamycin, penicillin-G, and tetracycline, but susceptible to oxacillin, TMP/SMX, and vancomycin. Identification of *S. pseudintermedius* was confirmed by MALDI-TOF mass spectrometry, as routine biochemical testing often misclassifies this organism as *S. aureus* or coagulase-negative *Staphylococcus*. The prescriber noted oxacillin susceptibility, and the patient completed a four-week course of amoxicillin-clavulanate, resulting in ulcer resolution within two months.

Over the next year, the patient experienced multiple episodes of recurrent bilateral LE edema and ulcerations. Trauma to the left leg led to swelling and yellowish drainage. A 21-day course of amoxicillin-clavulanate was prescribed empirically. Two months later, he self-reported recurrent bilateral leg ulcers with drainage and received two consecutive 21-day courses of amoxicillin-clavulanate. No wound cultures or physician notes were documented at the time of the refill.

**Figure 1:**
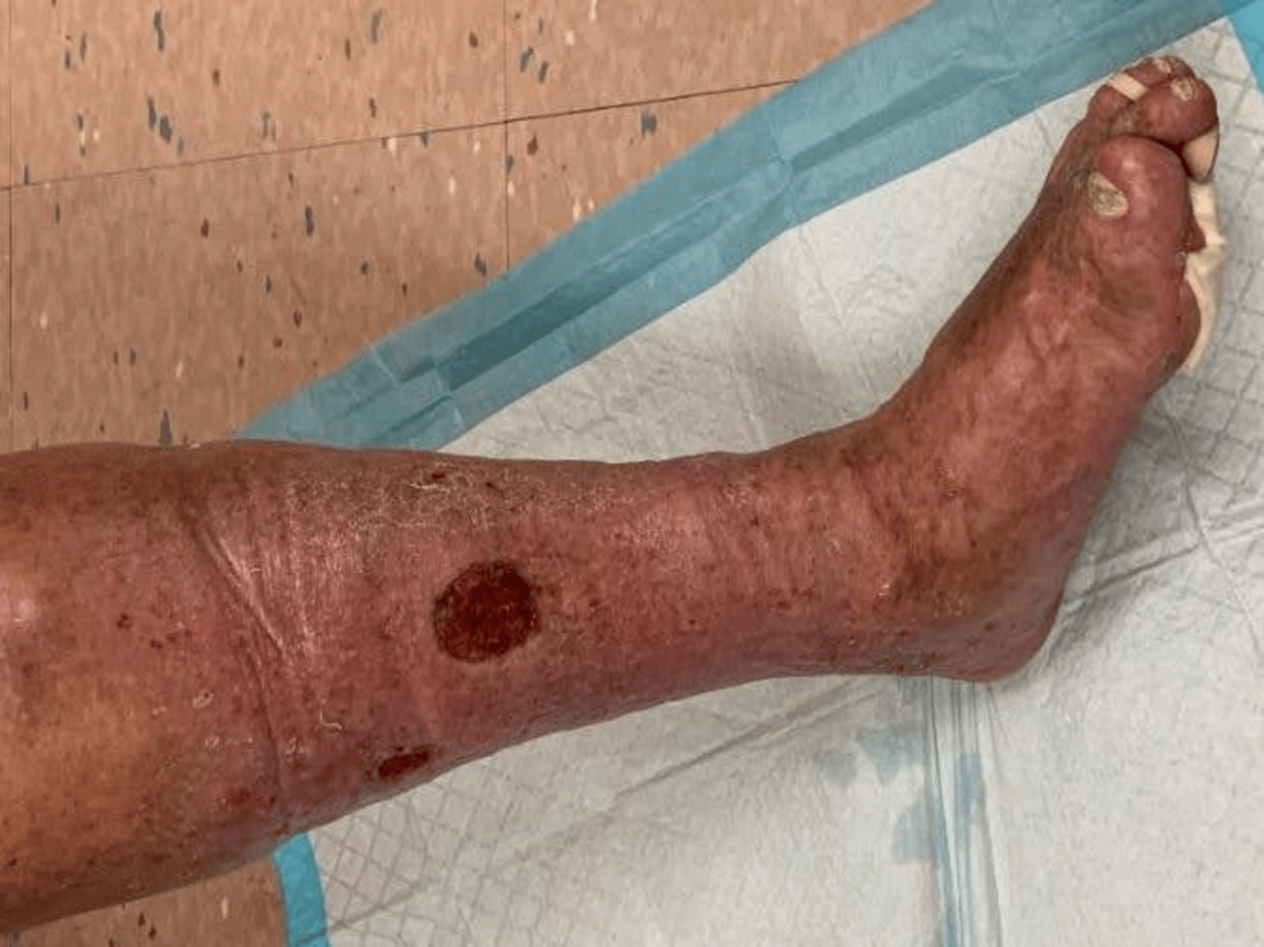
Left lower extremity ulcer with granulation tissue and surrounding erythema.

**Figure 2:**
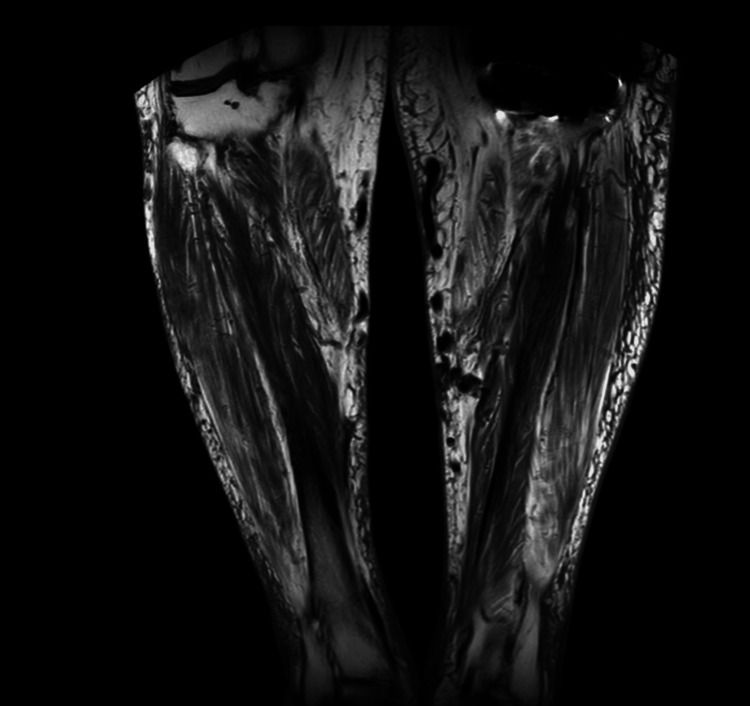
MRI T1-weighted images of the bilateral lower extremities (without contrast) demonstrate superficial soft tissue ulceration at the anterior mid-left leg, with diffuse subcutaneous soft tissue swelling and edema. No evidence of osteomyelitis was identified.

**Figure 3:**
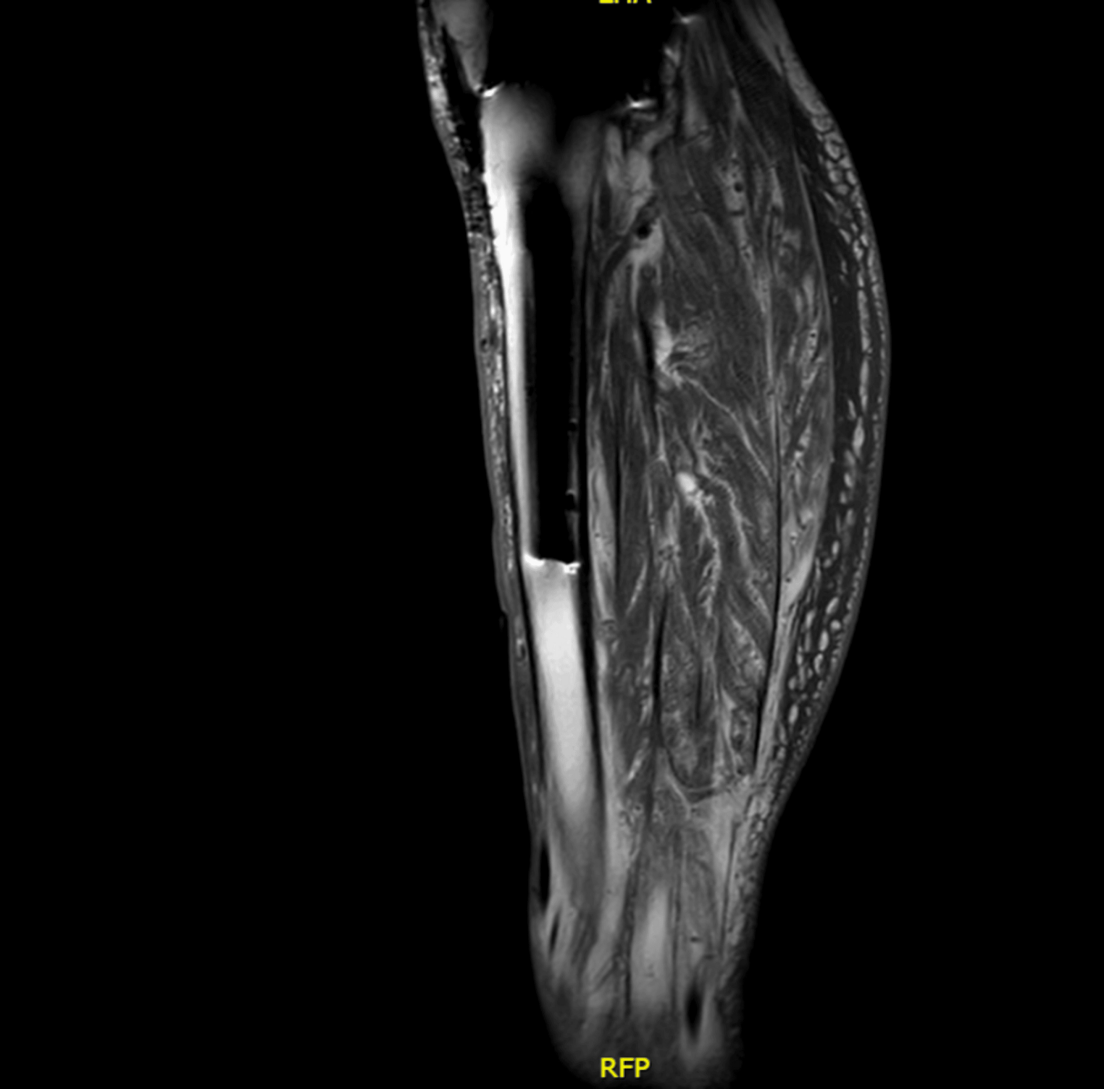
MRI STIR sequence of the left leg showing diffuse hyperintensity in the subcutaneous tissue consistent with edema. STIR, short tau inversion recovery; RFP, right-foot-posterior (orientation marker)

Two months later, he returned with a painful, burning, quarter-sized ulcer with granulation tissue but no purulence (Figure 1). MRI of the lower legs demonstrated superficial soft tissue ulceration at the anterior mid-left leg, with diffuse subcutaneous soft tissue swelling and edema, but there was no evidence of osteomyelitis identified (Figure 2 and Figure 3). Wound cultures grew *Citrobacter braakii, Enterococcus faecalis*, and* S. pseudintermedius* (resistant to penicillin-G, otherwise pansensitive). As there were no overt signs of infection, antimicrobial therapy was withheld. In the following month, he started TMP/SMX based on culture sensitivities, but discontinued the medication after three days due to diarrhea. Wound care with regular cleaning and debridement continued.

A subsequent culture again grew *S. pseudintermedius* and *Proteus mirabilis*. The *Proteus* isolate was intermediate to imipenem and resistant to tetracycline. The *S. pseudintermedius*, initially misreported as a coagulase-negative *Staphylococcus*, remained resistant only to penicillin-G.

**Figure 4:**
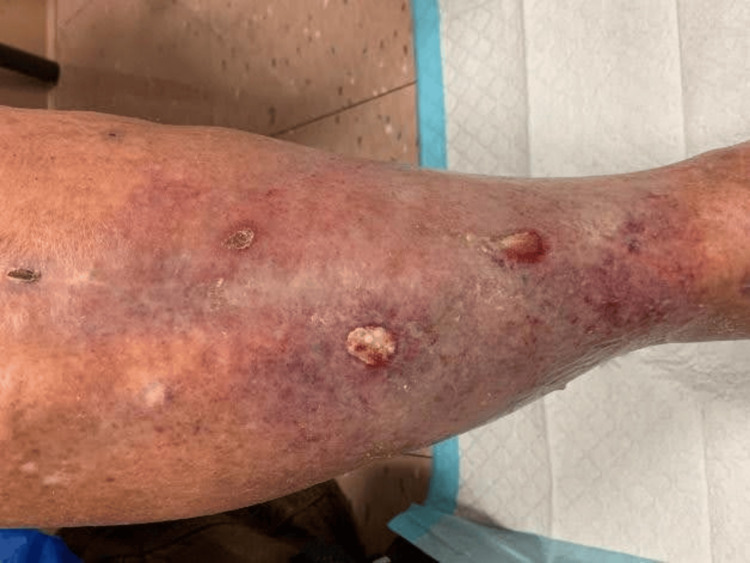
Recurrent ulceration of the right lower extremity with drainage and increased erythema before TMP/SMX treatment. TMP/SMX, trimethoprim/sulfamethoxazole

**Figure 5:**
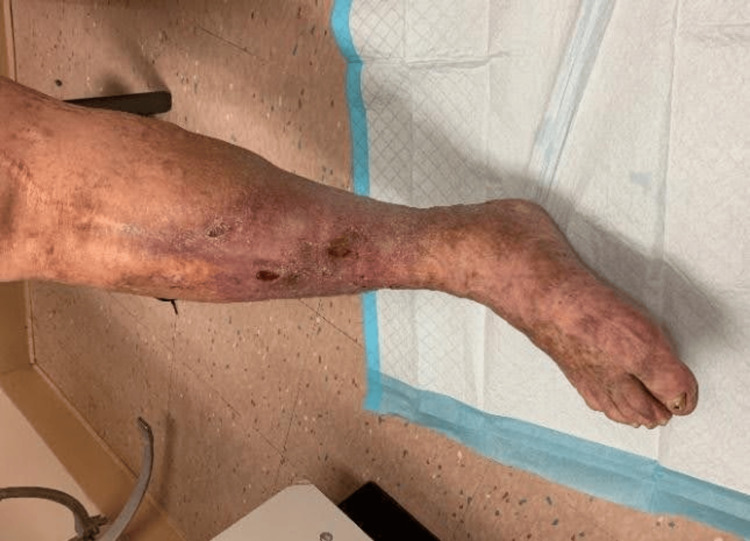
Right lower extremity ulcer showing improvement in size and surrounding erythema after initiating TMP/SMX treatment. TMP/SMX, trimethoprim/sulfamethoxazole

Three months later, the patient presented with 3x3 cm draining ulcers and increased LE erythema (Figure 4). Initial wound cultures were negative, but repeat cultures grew *S. pseudintermedius* and *Proteus mirabilis*. He started on doxycycline 100 mg twice daily for two weeks, which reduced drainage and inflammation. Resistance testing now showed *S. pseudintermedius* resistant to ciprofloxacin, levofloxacin (intermediate), erythromycin, tetracycline, and penicillin-G. Based on these results, his antibiotic regimen was switched to TMP/SMX. At follow-up, clinical improvement was noted (Figure 5), and the decision was made to continue TMP/SMX with close monitoring.

Around this time, the patient mentioned that his dog had recently passed away, though the cause of death was unspecified. No wounds or infections in his dog were reported. Over the following year, the ulcers gradually healed, and TMP/SMX was discontinued.

However, within the next two months, the patient returned with new ulcer drainage after reportedly sitting outdoors by a lake. The wounds progressively worsened, reaching up to 3.5x2.7 cm despite regular vascular surgery follow-up, repeated wound debridement, and wound care. No additional wound cultures were obtained during this period; therefore, the causative pathogen was unknown throughout this recurrence and could be due to a new infection from a microbe other than *S. pseudintermedius*. A year later, he started Natrox (continuous topical oxygen therapy), which led to significant improvement. Within a month, the ulcer had reduced to less than 1 cm in diameter. This patient’s recurrent skin infections finally achieved resolution after a course of seven years, with no recurrences reported on follow-up within the next year.

## Discussion

*S. pseudintermedius* is a coagulase-positive *Staphylococcus* commonly found as part of the normal skin and mucosal flora in dogs, where it acts as both a commensal organism and an opportunistic pathogen. In humans, it is an emerging zoonotic agent capable of causing a range of infections, most commonly SSTIs, but also occasionally invasive disease [3-6]. This case highlights the clinical relevance of *S. pseudintermedius* as a human pathogen and underscores the potential for household pets to serve as reservoirs.

This case demonstrates the importance of obtaining a detailed history of exposure, including pet ownership, especially in patients with recurrent SSTIs. The identification of *S. pseudintermedius* is often missed due to limitations in routine microbiologic methods, as it can be misidentified as *S. aureus* or *S. intermedius* using standard biochemical assays. Tools like MALDI-TOF or PCR can help identify the exact species more accurately, leading to better infection control and antibiotic treatment [8].

Early and accurate identification of *S. pseudintermedius* is crucial to preventing ongoing transmission and improving clinical outcomes. In our patient’s case, the pathogen was identified through wound culture, and treatment was initiated based on susceptibility results. Despite this, the patient’s complex medical history, including chronic venous insufficiency, diabetes risk, and poor wound healing, likely contributed to recurrent infections. Disruptions to health services during the COVID-19 pandemic limited patients' access to in-person consultations and timely follow-up appointments. Additionally, ongoing close contact with his dog may have facilitated repeated reinoculation with the organism.

Raising awareness among clinicians, microbiologists, and veterinarians about the zoonotic potential of *S. pseudintermedius* is essential. Such awareness could guide decisions regarding treatment, decolonization strategies, and even temporary separation from pets in certain high-risk scenarios. Other possible measures to prevent exposure include maintaining good hand hygiene, avoiding contact of wounds with a pet’s saliva, nasal secretions, or feces, and routine cleaning of pet bedding.

There are notable limitations in the evaluation of a patient’s exposure to a potential animal source. Often, veterinary assessments of pets are based on owner-provided summaries, and microbiological cultures are not performed on the animal. Veterinary medical records are often inaccessible or not pursued entirely, which may hinder a comprehensive exposure assessment. Furthermore, the emergence and persistence of resistant strains in domestic animals are a possibility considering antimicrobial use in humans and household pets [3-5,7].

The role of direct culture of pet flora and susceptibility testing in confirming zoonotic transmission and guiding treatment warrants further investigation. The potential benefits of isolation or decolonization measures should also be explored while carefully considering their practicality and emotional impact. In this case, the use of advanced identification methods enabled early detection and susceptibility-guided treatment, which proved critical to the patient’s clinical improvement.

## Conclusions

In summary, this case demonstrates how a typically non-pathogenic form of Staphylococcus in animals can result in recurrent, unresolved SSTIs in a human host. We acknowledge that the infection described in our report was likely acquired through direct inoculation from casual contact with the patient’s domesticated canine. However, a lack of microbiological testing of the dog prevents definitive confirmation of zoonotic transmission. Given the potential for antimicrobial resistance and the availability of modern diagnostic techniques, clinicians should consider that coagulase-positive Staphylococcus infections, previously presumed to be S. aureus, may in fact involve S. pseudintermedius, as seen in this patient.

Although S. pseudintermedius was initially overlooked, advances in accessible laboratory methods enabled species-level identification and clarified the true causative pathogen. These findings were critical for selecting appropriate antimicrobial therapy and achieving eradication of the infection. Moving forward, clinicians should conduct careful assessments of risk factors such as pet ownership and direct contact with canine saliva or secretions, which may serve as transmission routes for zoonotic pathogens. Therefore, in cases where a patient’s history and risk of exposure are clearly documented, consideration of S. pseudintermedius as a potential causative pathogen in SSTIs is justified and should not be overlooked. Transmission from the dog is plausible but cannot be definitively confirmed due to limitations in the evidence.
